# Drug-Drug Interaction Potential, Cytotoxicity, and Reactive Oxygen Species Production of *Salix* Cortex Extracts Using Human Hepatocyte-Like HepaRG Cells

**DOI:** 10.3389/fphar.2021.779801

**Published:** 2021-11-18

**Authors:** João Victor Dutra Gomes, Corinna Herz, Simone Helmig, Nadja Förster, Inga Mewis, Evelyn Lamy

**Affiliations:** ^1^ Molecular Preventive Medicine, University Medical Center and Faculty of Medicine—University of Freiburg, Freiburg, Germany; ^2^ Institute for Occupational and Social Medicine and Department of Anesthesiology, Justus-Liebig University Giessen, Giessen, Germany; ^3^ Division Urban Plant Ecophysiology, Humboldt-Universität zu Berlin, Berlin, Germany

**Keywords:** *Salix* species, willow bark, CYP450 enzymes, drug interaction, herb-drug interaction

## Abstract

Herbal preparations of willow bark (*Salix* cortex) are available in many countries as non-prescription medicines for pain and inflammation, and also as dietary supplements. Currently only little information on toxicity and drug interaction potential of the extracts is available. This study now evaluated the effects of two *Salix* cortex extracts on human hepatocyte-like HepaRG cells, in view of clinically relevant CYP450 enzyme activity modulation, cytotoxicity and production of reactive oxygen species (ROS). Drug metabolism via the CYP450 enzyme system is considered an important parameter for the occurrence of drug-drug interactions, which can lead to toxicity, decreased pharmacological activity, and adverse drug reactions. We evaluated two different bark extracts standardized to 10 mg/ml phenolic content. Herein, extract S6 (*S. pentandra*, containing 8.15 mg/ml total salicylates and 0.08 mg/ml salicin) and extract B (industrial reference, containing 5.35 mg/ml total salicylates and 2.26 mg/ml salicin) were tested. Both *Salix* cortex extracts showed no relevant reduction in cell viability or increase in ROS production in hepatocyte-like HepaRG cells. However, they reduced CYP1A2 and CYP3A4 enzyme activity after 48 h at ≥25 μg/ml, this was statistically significant only for S6. CYP2C19 activity inhibition (0.5 h) was also observed at ≥25 μg/ml, mRNA expression inhibition by 48 h treatment with S6 at 25 μg/ml. In conclusion, at higher concentrations, the tested *Salix* cortex extracts showed a drug interaction potential, but with different potency. Given the high prevalence of polypharmacy, particularly in the elderly with chronic pain, further systematic studies of *Salix* species of medical interest should be conducted in the future to more accurately determine the risk of potential drug interactions.

## Introduction

The bark of various willow (*Salix* cortex) varieties has long been used in medicine against inflammation, as a painkiller and against fever (Wood, 2015). Recently, our group demonstrated the anti-inflammatory potential of *Salix* cortex extracts in SARS-CoV-2 peptide and bacterial lipopolysaccharide (LPS)-activated human *in vitro* systems, providing new evidence of the potential usefulness of *Salix* products as therapeutics (Le et al., 2021). *Salix* cortex is also used in dietary supplements, e.g., for weight reduction and to enhance performance in sports (Matyjaszczyk and Schumann, 2018). Despite its long history of use, so far only little data is available on the toxicity or potential adverse effects of *Salix* cortex preparations (Shara and Stohs, 2015). Based on a limited number of studies, one safety report by Shara and Stohs (2015) recommended that people who 1) are allergic to aspirin, 2) suffer from pathological conditions such as gastritis, stomach ulcers, diabetes, asthma, or hemophilia; or 3) are under anticoagulant-drug therapy, as well as beta-blockers, diuretics, and non-steroidal anti-inflammatory drugs (NSAIDs), may avoid *Salix* cortex.

Herbal preparations including *Salix* cortex extracts contain hundreds of phytochemicals that can act in different ways, encompassing the risk of drug interactions that is the ability to modify the action or effect of another drug administered successively or simultaneously. Considering that the usage of pain medication plays a major role in polypharmacy (Marengoni et al., 2014; Schneider et al., 2021; Young et al., 2021), knowledge of the interaction potential of *Salix* species is of great importance, as this could help avoid drug-related problems that could affect patients’ safety. An important determinant in the occurrence of drug interaction is the drug metabolism via the cytochrome P450 (CYP450) system (Guengerich, 2008). This class, which is predominantly expressed in the liver, has more than 50 enzymes, but from these, only six of them (CYP1A2, CYP2C9, CYP2C19, CYP2D6, CYP3A4, and CYP3A5) metabolize 90 percent of the medications (Wilkinson, 2005). Considering the historical relevance of *Salix* cortex extracts in traditional medicine, their wide commercial availability, as well as their potential new pharmacological application, in the present study we investigated the potential of Salix *cortex* extracts for drug-drug interactions with respect to CYP450 enzymes relevant to drug metabolism. For this purpose, we used the human hepatocyte-like cell line HepaRG. As a validated *in vitro* model to investigate drug effects on metabolism enzymes, the HepaRG cell line is considered an alternative to primary *ex vivo* cultured human hepatocytes, especially in studies related to detoxification metabolism, such as CYP450 enzyme activities for predicting drug-drug interaction (Aninat et al., 2006; Anthérieu et al., 2010). Potential cytotoxicity was then assessed by measuring adenosine triphosphate (ATP) and lactate dehydrogenase (LDH), and oxygen radical formation was measured by electron magnetic resonance spectroscopy (EPR) in the cells.

## Materials and Methods

### Chemicals and Reagents

Fetal calf serum (FCS), L-glutamine and phosphate buffered saline (PBS, without Ca and Mg), penicillin-streptomycin (P/S) solution, L-glutamine solution, RPMI-1640, DMEM, William’s Medium E + GlutaMAX™, human recombinant insulin zinc solution (4 mg/ml), trypsin-EDTA 10x (5 mg/ml and 2.2 mg/ml), Trypsin (0.5%) solution and phosphate buffered saline (PBS, without Ca^2+^ and Mg^2+^), and penicillin/streptomycin solution (10,000 U/ml and 10,000 μg/ml) were purchased from Gibco™, Life Technologies GmbH (Darmstadt, Germany). Hydrocortisone 21-hemisuccinate sodium salt, omeprazole (≥99.0%), rifampicin (≥97%), ketoconazole (99%), naringenin (98%), menadione (≥98.0%), and acetylsalicylic acid (100%) were purchased from Sigma-Aldrich (Taufkirchen, Germany). Troglitazone (≥98%) was purchased from Santa Cruz (Heidelberg, Germany). 1-hydroxy-3-methoxy-carbonyl-2,2,5,5-tetramethylpyrolidine hydrochloride (CMH), diethyldithio-carbamate trihydrate (DETC), deferoxamine (DFO), and Krebs-HEPES buffer were purchased from Noxygen Science Transfer & Diagnostics GmbH, Elzach, Germany).

### Cell Culture

The human hepatic cell line HepaRG was obtained from Biopredic International® (Rennes, France). The cell line was cultured in William’s Medium E + GlutaMAX™, supplemented with 10% FCS, 100 U/mlµ penicillin, and 100 μg/ml streptomycin, 50 µM hydrocortisone 21-hemisuccinate sodium salt, and 5 μg/ml human insulin. The maintenance and differentiation of the cell line was performed according to Biopredic International® instructions, as previously described (Le et al., 2021). Cells were maintained at 37°C in a humidified incubator with a 5% CO_2_ and 95% air atmosphere.

### *Salix* Cortex Extract Preparations

*Salix* cortex extracts were prepared and standardized as previously described (Le et al., 2021). *S. pentandra* clone PE1 (extract S6), originally collected in 2006 in Eggersdorf (Brandenburg, Germany), was cultivated in Wriezen (in northeastern Berlin, Brandenburg, Germany). One-year-old branches of the clone were cut off in August 2016 and bark was peeled at a height from 10–100 cm. Afterwards, the bark material was frozen (−80°C) and immediately lyophilized. Hardwood cuttings of PE1 were also planted in a clone collection at Humboldt-Universität zu Berlin (Germany) to guarantee the availability and conservation of the *Salix* clone. The bark was extracted using a solution of 70% methanol and 0.1% formic acid. Extract B refers to a willow bark reference used for phytopharmaceutical production, which was provided from Bionorica SE (Neumarkt, Germany). Both extracts (B and S6) were standardized to 10 mg/ml phenolic content using high performance liquid chromatography (HPLC). Based on the reported pharmacological potential and knowledge of characteristic compounds in different Salix species, the following phytochemicals were used to standardize the extracts: salicylates (salicin, salicortin, 2′-O-acetylsalicin, 2′-O-acetylsalicortin, and tremulacin), flavan-3-ols (catechin and epicatechin), flavonoids (two isomers of naringenin-5-*O*-glucoside, naringenin-7-*O*-glucoside, luteolin-7-*O*-glucoside, quercetin-hexoside, and isosalipurposide), other phenolic compounds (triandrin, two caffeic acid derivatives, and syrengin). S6 extract contained 8.15 mg/ml total salicylates and 0.08 mg/ml salicin, and extract B contained 5.35 mg/ml total salicylates and 2.26 mg/ml salicin (Le et al., 2021).

### Assessment of Cell Viability and Cytotoxicity

The enzyme lactate dehydrogenase (LDH) is involved in energy production and found in almost all cells of the human body. Upon damage, it is released from the cell in the medium and can thus be used as a marker for cytotoxicity. After 24 h extract exposure, LDH was quantified using an LDH-Glo™ Cytotoxicity Assay kit (Promega GmbH, Mannheim, Germany) according to the manufacturer’s instructions. As positive control, cell exposure to 0.2% triton-X for 15 min was used.

Adenosine triphosphate (ATP) is a key indicator of cellular activity and has been used as another marker of cytotoxicity upon 24 and 48 h treatment using the CellTiter-Glo® 2.0 Cell Viability Assay (Promega GmbH, Mannheim, Germany) according to the manufacturer’s instructions. As positive control, cell exposure to 0.2% triton-X for 24 or 48 h was used. In both assays, 0.5% distilled water was used as solvent control (SC).

### Assessment of Reactive Oxygen Species (ROS) Production Using EPR

The production of reactive oxygen species (ROS) by *Salix* cortex extracts in hepatocyte-like HepaRG cells was detected using electron paramagnetic resonance (EPR) spectroscopy as described by Lamy et al. (2013) and adapted by Odongo et al. (2017). Differentiated HepaRG cells were treated with different concentrations of *Salix* cortex extracts or 0.5% distilled water (solvent control) for 1 or 24 h. Cell exposure to 200 µM menadione for 30 min was used as positive control. Afterwards, for ROS detection, 200 µM 1-hydroxy-3- methoxy-carbonyl-2,2,5,5-tetramethylpyrolidine hydrochloride (CMH, Noxygen Science Transfer & Diagnostics GmbH, Elzach, Germany), 25 µM deferoxamine (DFO), and 5 µM DETC were used in Krebs-HEPES buffer for 30 min (Odongo et al., 2017). Supernatants were then measured by EPR spectroscopy for ROS production evaluation. The instrument setting and the number of scans used were set as previously described (Lamy et al., 2013).

### Cytochrome P450 Enzyme Activity Quantification

The effects of *Salix* cortex extracts on CYP1A2 and CYP3A4 enzyme activity were evaluated at 1 and 48 h treatment (Bernasconi et al., 2019; FDA, 2020) using the cell based P450-Glo™ Induction/Inhibition Assay Systems (Promega, Walldorf, Germany) according to the manufacturer’s protocol. In brief, HepaRG cells were differentiated as described above in white 96 well plates. After differentiation, cells were incubated for 1 or 48 h with the *Salix* cortex extracts, or 0.5% distilled water (solvent control). For 48 h treatment, the medium was exchanged after 24 h with the addition of fresh extract. To analyze enzyme activity of CYP2C19, the biochemical P450-Glo™ CYP2C19 Assay and Screening System (Promega, Walldorf, Germany) was used according to the manufacture’s protocol.

50 µM omeprazole (induction) and 320 µM naringenin (inhibition) were used as positive controls (PC) in the CYP1A2 assay.10 µM Rifampicin (induction) and 10 µM ketoconazole (inhibition) were used as positive control in the CYP3A4 assay. 10 μg/ml (22.6 µM) troglitazone was used as positive control for CYP2C19 inhibition (Wishart et al., 2018).

### Quantitative PCR for CYP450 mRNA Expression

CYP2C19 mRNA expression was quantified using qRT-PCR. In brief, differentiated HepaRG cells were treated with different concentrations of *Salix* cortex extracts or 0.5% distilled water (solvent control) for 6 or 48 h. Total RNA from HepaRG cells was isolated using the RNeasy mini Isolation kit from Qiagen (Hilden, Germany) followed by DNA purification step using the RNase-free DNase kit from Qiagen (Hilden, Germany) according to the manufacturer’s instructions. RNA quality and quantity were measured using a NanoDrop ND-1000 spectrophotometer (Thermo Scientific, Freiburg, Germany). Isolated RNA was resuspended in 10 µL of RNAse-free water. Each sample was treated twice with 2 µL RNAse-free DNAse 1unit/μL (Qiagen, Hilden, Germany) for 10 min at 37°C to eliminate remaining DNA. The prepared RNA was reverse-transcribed as previously described (Helmig et al., 2009). For quantitative comparison of CYP2B6, CYP2C19 and CYP2D6 mRNA levels real-time PCR was performed using SYBR-green fluorescence in a LightCycler^®^ System (Roche Diagnostic GmbH). After optimization of PCR conditions, amplification efficiency was tested in standard curves using serial cDNA dilutions. The correlation coefficient had to be above 0.9 and the slope around −3.5. Amplification specificity was checked using melting curves. Gene expression was related to the mean expression of the three housekeeping genes (HSK) beta-2-microglobulin (B2M), hypoxanthine-guanine phosphoribosyltransferase (HPRT) and glycerinaldehyd-3-phosphat-dehydrogenase (GAPDH) (Vandesompele et al., 2002). Calculations of expression was performed with the 2^−ΔΔCT^ method (Pfaffl, 2001). The sequences of the used specific primers are listed in Table 1 (Zhang et al., 2005; Chen et al., 2014).

**TABLE 1:** Sequences of specific primers.

Primer	Sequence	GeneBank	Reference
Cyp2B6	For 5′-CCA​GCT​TCC​GAG​GGT​ACA​TC-3′	NM_000767.5	NCBI Blast Primer
	Rev 5′-CAG​GAT​TGA​AGG​CGT​CTG​GT-3′		
Cyp2C19	For 5′-CAA​CAA​CCC​TCG​GGA​CTT​TA-3′	NM_000769	Chen et al. (2014)
	Rev 5′-GTC​TCT​GTC​CCA​GCT​CCA​AG-3‘		
Cyp2D6	For 5′-TTC​CTG​CCT​TTC​TCA​GCA​GG-3′	NM_00106.5	NCBI Blast Primer
	Rev 5′-ACC​GAG​AAG​CTG​AAG​TGC​TG-3′		
B2M	For 5′-ACT​GAA​TTC​ACC​CCC​ACT​GA-3′	M17987	Zhang et al. (2005)
	Rev 5′-CCT​CCA​TGA​TGC​TGC​TTA​CA-3′		
HPRT	For 5′-ATG​CTG​AGG​ATT​TGG​AAA​GGG-3′	NM_000194.2	NCBI Blast Primer
	Rev 5′GCA​CAC​AGA​GGG​CTA​CAA​TG-3′		
GAPDH	For 5′TGC​ACC​ACC​AAC​TGC​TTA​GC-3′	NM_002046	NCBI Blast Primer
	Rev 5′GGC​ATG​GAC​TGT​GGT​CAT​GAG-3		

PCR reactions were carried out in a final volume of 20 µl using 1x ABsolute® QPCR SYBR Green Capillary Mixes (Abgene, Brumath, France), 300 nM of primers and 2 µL cDNA. The PCRs started with a 15 min denaturation phase and at the end, a melting curve was acquired form 40°C to 95°C at a thermal transition rate of 0.1°C for 40 s. Specific conditions for primers were as follows: CYP2B6 45 cycles of 95°C for 10 s, 58°C for 15 s, 72°C for 15 s; CYP2C19 45 cycles of 95°C for 15 s, 61°C for 15 s, 72°C for 20 s; CYP2D6 45 cycles of 95°C for 10 s, 61°C for 15 s, 72°C for 15 s. The PCR conditions for HSKs were as follows: B2M 55 cycles of 95°C for 10 s, 63°C for 10 s, 72°C for 25 s; HPRT 45 cycles of 95°C for 15 s, 61°C for 15 s, 72°C for 15 s; GAPDH 45 cycles of 95°C for 10 s, 61°C for 10 s, 72°C for 25 s. All measurements were made without information about the origin of the samples and were performed in duplicate.

### Statistical Analysis

Data were analyzed using GraphPad Prism 6.0 software (La Jolla, CA, United States) and presented as means + SD of at least three independent experiments. When comparing multiple means, the results were analyzed either by one-way ANOVA followed by Dunnett’s multiple comparison tests or two-way ANOVA followed by Tukey’s multiple comparison test.

## Results

### CYP450 Enzyme Activity Quantification

**FIGURE 1:**
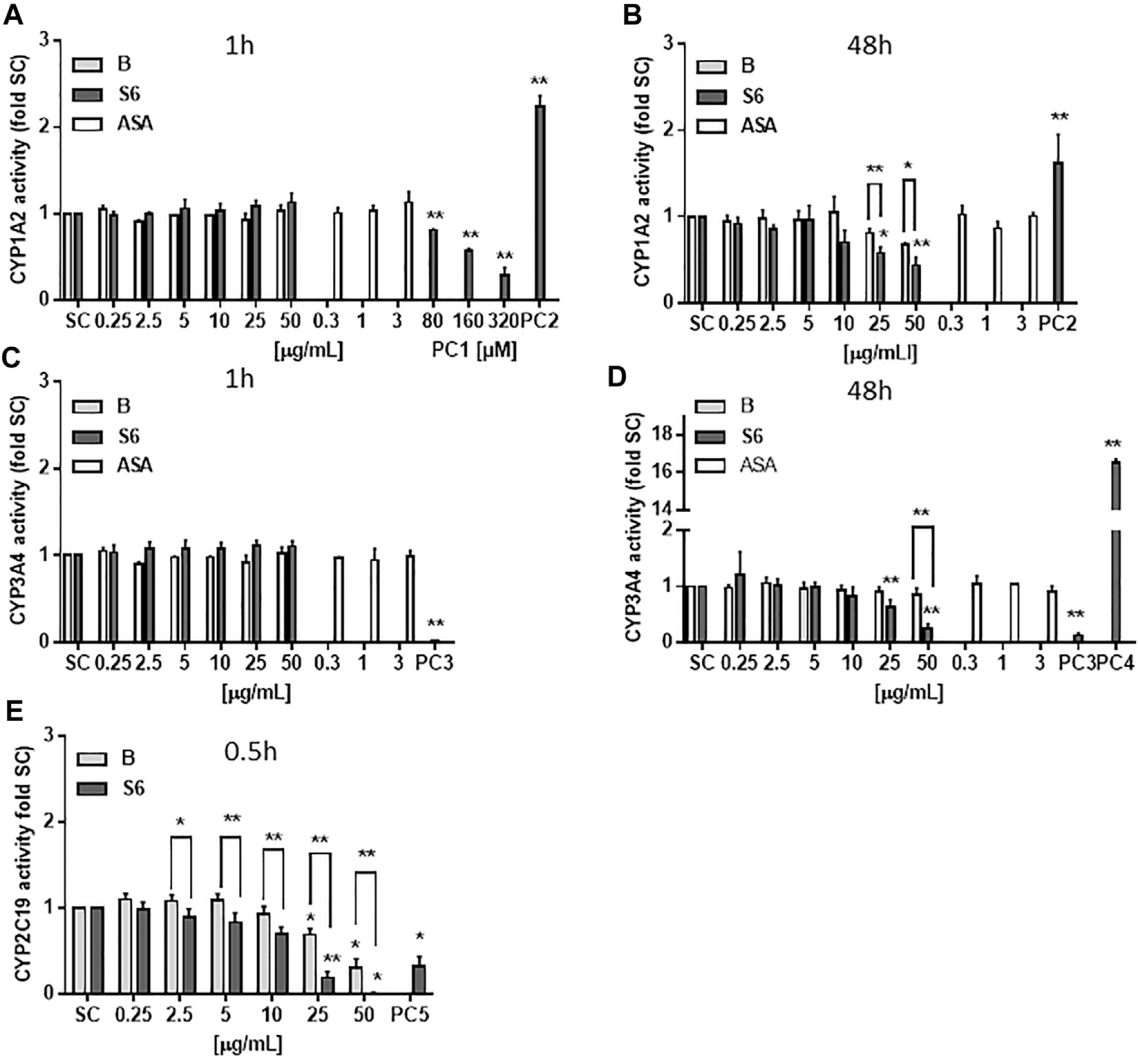
CYP450 enzyme activity quantification after treatment with *Salix* cortex extracts using a luminescent method. **(A–D)** Differentiated HepaRG cells were exposed to extracts for 1 or 48 h before analysis. **(E)** CYP2C19 enzyme activity was analysed in a cell-free assay after 0.5 h incubation of extracts with a human recombinant CYP2C19 enzyme, followed by analysis. Positive control (PC): 320 μM naringenin (CYP1A2 inhibition, PC1), 50 μM omeprazole (CYP1A2 induction, PC2), 10 μM rifampicin (CYP3A4 inhibition, PC3), 10 μM ketoconazole, (CYP3A4 induction, PC4), and 22.6 μM troglitazone (CYP2C19 inhibition, PC5). ASA, acetylsalicylic acid. The values are presented as means + SD (CYP1A2 1 and 48 h, n = 3; CYP3A4 1 h, n = 3, 48 h n = 4; CYP2C19 n = 3). Ordinary one-way ANOVA was used for statistical analysis, followed by a Dunnett test. Significance was evaluated between extracts and solvent control (a. d.) as well as between extract S6 and B. *p > 0.05, **p > 0.01.

The experiments tested a concentration range of the extracts that had previously shown pharmacological activity in terms of blocking LPS-induced inflammation in primary human immune cells (Le et al. 2021). To investigate potential drug interaction, CYP450 enzyme activity of three enzymes (CYP1A2, CYP3A4 and CYP2C19) was quantified upon *Salix* cortex extract exposure. As shown in Figure 1A, narigenin (PC1) reduced CYP1A2 enzyme activity in hepatocyte-like HepaRG cells after 1 h by 70%. Omeprazole (PC2) increased the CYP1A2 enzyme activity after 1 h by more that 2-fold (1.6 at 48 h, Figures 1A,B). The *Salix* cortex extracts did not affect cellular enzyme activity at that time. After 48 h treatment, both extracts reduced the enzyme activity at high concentrations (25 or 50 μg/ml), while the effect was more pronounced by S6, then. Ketoconazole (PC3) completely abolished CYP3A4 activity after 1 h exposure of HepaRG cells, while rifampicin (PC4) triggered enzyme activity induction by about 16-fold compare to control after 48 h (Figures 1C,D). Acetylsalicylic acid (ASA) did not affect CYP1A2 and CYP3A4 enzyme activity after 1 or 48 h at the tested concentrations (Figures 1A–D). For assessment of CYP2C19 enzyme activity, a cell-free assay was used. After 0.5 h, troglitazone (PC5) reduced CYP2C19 activity by 68%; at ≥25 μg/ml both *Salix* cortex extracts also reduced enzyme activity by 81% (extract S6) and 31% (extract B) compared to solvent control. Again, the inhibitory effect of S6 on CYP450 enzyme activity was stronger compared to extract B.

### CYP2C19 mRNA Expression

**FIGURE 2:**
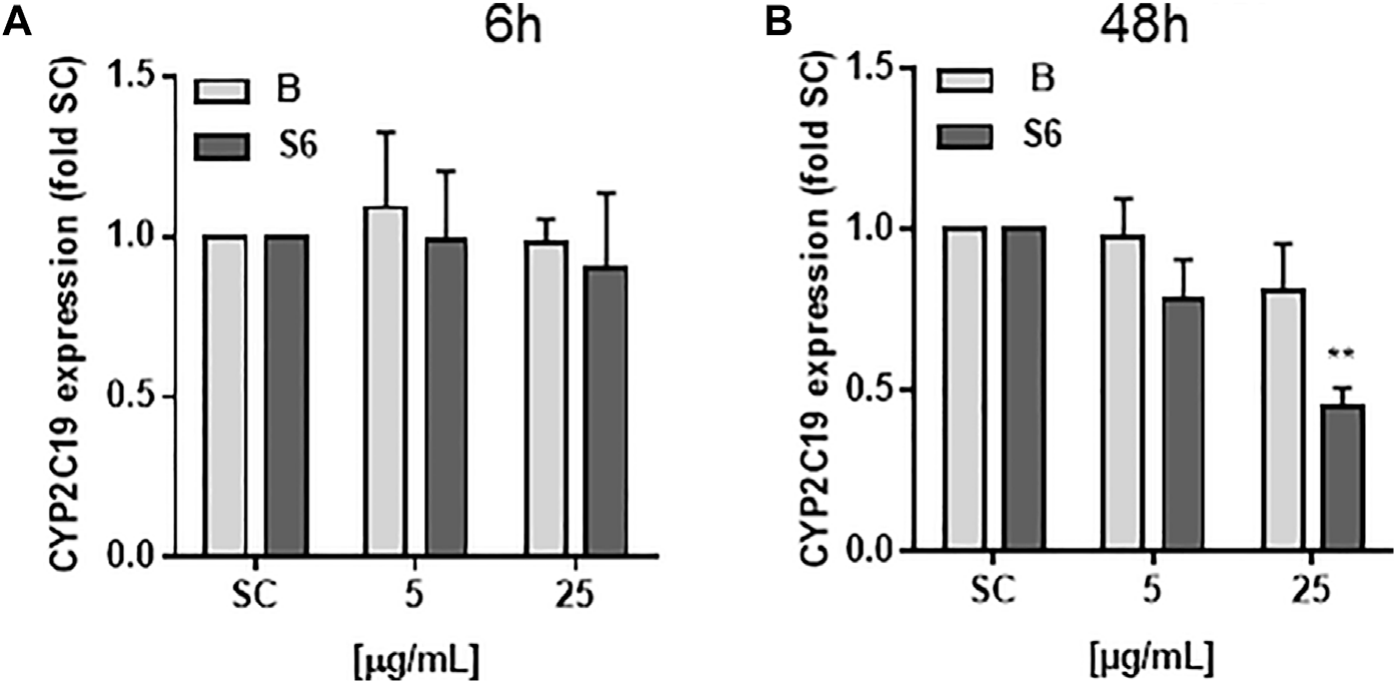
mRNA levels of CYP2C19 in differentiated HepaRG cells after *Salix* cortex extracts treatment. mRNA levels were quantified by qRT-PCR. Differentiated HepaRG cells were treated with *Salix* cortex extracts (extract B or S6) by 6 h **(A)** or 48 h **(B)**. CYP2C19 expression levels were expressed as mean + SD; (*n* = 3; S6, 48 h *n* = 2). Ordinary one-way ANOVA was used for statistical analysis, followed by a Dunnett test versus solvent control (SC: 0.5% destilled water) group. **p < 0.001.

Differentiated HepaRG cells were exposed for 6 and 48 h to *Salix* cortex extracts and mRNA expression of CYP2D6, CYP2B6 and CYP2C19 quantified using qRT-PCR (Figure 2). The baseline mRNA levels of CYP2D6 and CYP2B6 were very low in HepaRG cells and no mRNA expression upon treatment could be seen (data not shown). Baseline CYP2C19 mRNA levels were not reduced after 6 h treatment with *Salix* cortex extracts. After 48 h treatment with 25 μg/ml extract S6, but not B significantly reduced CYP2C19 mRNA expression by 55%.

### Cytotoxicity and ROS Production

**FIGURE 3:**
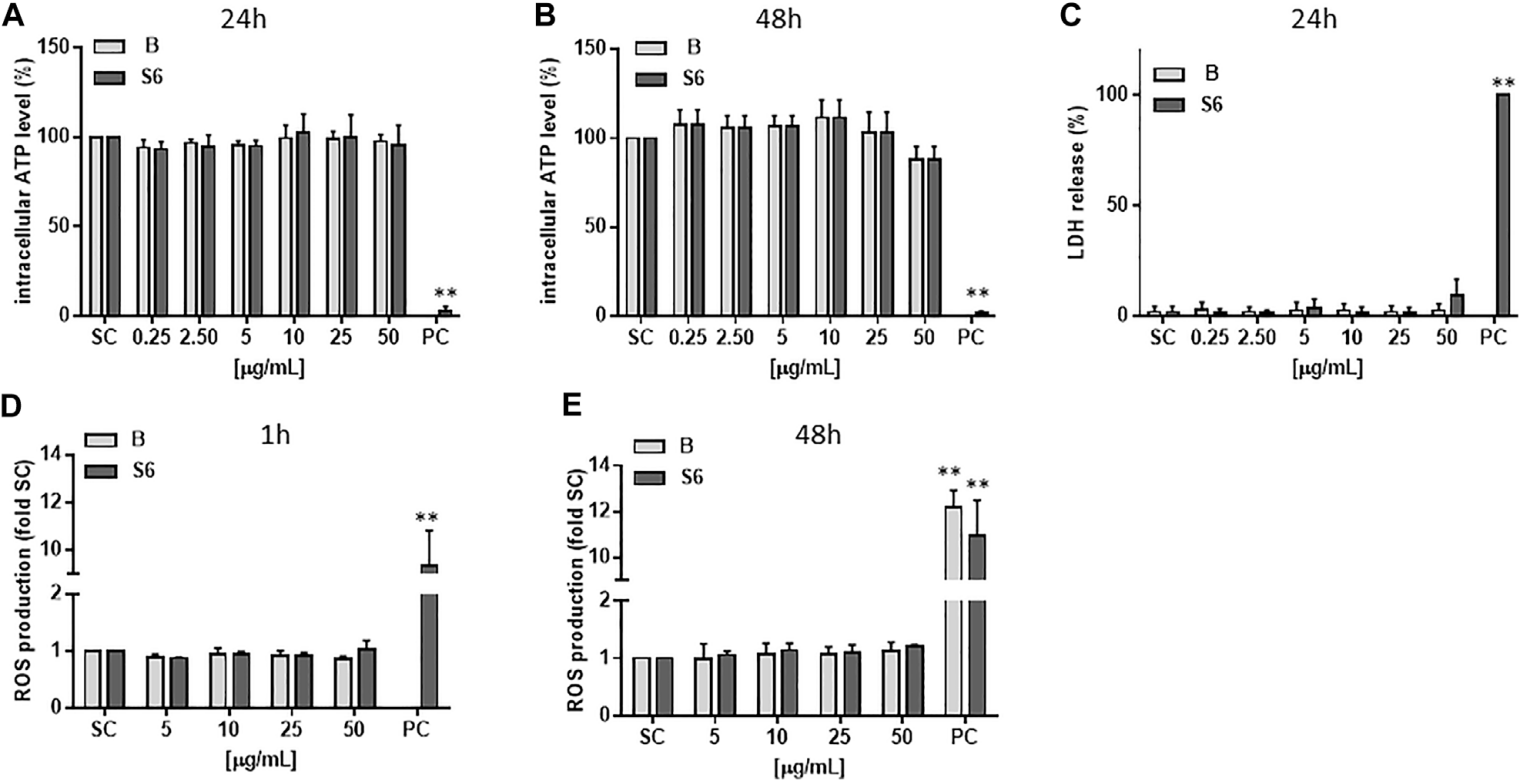
Cytotoxicity and ROS production of *Salix* cortex extracts in differentiated HepaRG cells. ATP levels were analysed after **(A)** 24 h **(B)** 48 h of extract treatment. n = 3 **(C)** LDH cell release was measured after 24 h extract treatment. 0.2% Triton-X was used as positive control (PC). n = 4 **(D, E)** ROS production was measured by EPR spectroscopy after **(D)** 1 h or **(E)** 24 h of extract treatment. 200 μM menadione for 30 min. was used as positive control (PC). n = 3. The values are presented as means + SD. Ordinary one-way ANOVA was used for statistical analysis, followed by a Dunnett test versus solvent control (SC: 0.5% destilled water) group. **p < 0.01.

As given in Figures 3A–C, neither of the two extracts affected intracellular ATP levels or triggered LDH release in hepatocyte-like HepaRG cells at the tested concentrations (0.25–50 μg/ml). We also tested whether the extracts could elevate the level of intracellular ROS in the cells, which in turn could cause damage to lipids, proteins and DNA. From Figures 3D,E it can be seen that after treatment with *Salix* cortex extracts for 1 or 48 h, no increase in ROS production could be detected.

## Discussion

The biggest consumers of prescription and over-the-counter medicines are older adults (Qato et al., 2008; Qato et al., 2016), and self-medication (Vacas Rodilla et al., 2009; Jerez-Roig et al., 2014) as well as consumption of non-prescription medicines, herbal and other dietary supplements in the first place, is widespread among them (Izzo and Ernst, 2009; de Souza Silva et al., 2014; Raji et al., 2017). Conditions of chronic pain, or other chronic conditions, such as diabetes, heart disease, stroke, or cancer, may experience concurrent use of multiple medications (Lunsky and Modi, 2018; Schneider et al., 2021). With the number of drugs, the risk of drug interactions increases exponentially while many drug interactions can be explained by changes in metabolic enzymes in the liver and other extrahepatic tissues. Interaction with hepatic CYP450 enzymes in terms of induction or inhibition is here one of the most important causes after co-administration of medications (Cadieux, 1989; Johnell and Klarin, 2007). CYP450 enzyme induction usually leads to accelerated biotransformation of a drug. For most drugs, accelerated metabolism results in decreased efficacy, but if a pro-drug is activated by CYP450 enzymes, its efficacy and/or toxicity may increase. When two drugs compete over the same enzyme receptor site, an enzyme inhibition occurs. The stronger inhibitor predominates, resulting in decreased metabolism of the competing drug. This can result in increased serum levels of the unmetabolized drug and thus greater potential for toxicity. For drugs whose pharmacological activity requires biotransformation from a pro-drug form, inhibition may result in decreased efficacy. Besides substrate competition, a drug can also reduce enzyme activity due to direct interaction or mRNA inhibition (Lee et al., 2009).

The drug interaction experiments reported in the present study were carried out using hepatocyte-like HepaRG cells. Excluding CYP2A6 and CYP2E1, the HepaRG cell line has been reported to express high functional levels of most of the major xenobiotic metabolizing CYP450 enzymes. These activities were then found to be inhibited and induced by prototypical compounds at comparable levels to primary hepatocytes (Turpeinen et al., 2009). With these characteristics, HepaRG cells have been proposed to be a more physiologically relevant pre-clinical platform for drug–drug interaction studies and safety pharmacology compared to e.g., the pre-clinically widely used cell line HepG2. Even though HepG2 cells are inexpensive and convenient, they lack a substantial set of liver-specific functions, particularly CYP450 activity (Gómez-Lechón et al., 2010). So far, there are only few reports on *Salix* cortex extracts investigating a CYP450 interaction potential. Using a cell-free fluorimetric *in vitro* assay, an ethanolic extract of *Salix planifolia* was found to inhibit CYP2C8 (60.9%), CYP2C19 (48.5%), CYP3A4 (92.3%), CYP3A5 (73.9%), and CYP3A7 (71.4%) at 10 μg/ml concentration. All other investigated enzymes were inhibited by less than 30.0%, which includes CYP1A2 (Tam et al., 2009). In HepaRG cells, we observed a low CYP1A2 and CYP3A4 interference potential of the tested *Salix* cortex extracts at a concentration which was about 5-fold higher as compared to an effective anti-inflammatory concentration reported earlier by us (Le et al., 2021). This effect was evident only after 48 h, which argues against a direct CYP enzyme activity interaction potential. CYP2C19 metabolizes important drugs in clinical practice, such as proton pump inhibitors (esomeprazole, lansoprazole, omeprazole, pantoprazole, rabeprazole), clopidogrel, tamoxifen, diazepam, citalopram, or escitalopram (Sienkiewicz-Oleszkiewicz and Wiela-Hojeńska, 2018). For this enzyme, our data suggest that both *Salix* cortex extracts have the potential to interfere with drug metabolism, as they both reduced CYP2C19 enzyme activity in a concentration-dependent manner after 30 min incubation. As with the other enzymes investigated, extract S6 was more potent in enzyme inhibition than extract B. On mRNA level, only S6 significantly reduced CYP2C19 expression in HepaRG cells. It is certain that none of the observed effects on CYP450 enzymes can be attributed to cytotoxic effects, since there was no reduction in ATP levels, increase in LDH or ROS production upon *Salix* cortex extract treatment in HepaRG cells. The two extracts differed in their salicylate content, which might account for the observed differences, but information on CYP450 regulation by e.g., acetylsalicortin or acetylsalicin, which were both present solely in extract S6, does not exist so far. In contrast to extract B, extract S6 also contained the flavonoids catechin (0.78 mg/ml) and epicatechin (0.03 mg/ml) (Le et al., 2021). For both compounds no relevant inhibition of CYP1A2, CYP2C9, CYP2D6, and CYP3A4 could be detected in a study on human liver microsomes (Satoh et al., 2016), which confirmed previous data (Muto et al., 2001). Thus, it is unlikely that the presence of these flavonoids add to the observed effects. For the aglycone of quercetin, some weak CYP450 activity inhibition has been described (Mohos et al., 2020). Quercetin-hexoside (but not the aglycone) is present in S6 at a 3-fold higher concentration compared to extract B. In contrast, extract B contains some O-glucosides of naringenin. For the aglycone CYP1A2 inhibition has been reported by Fuhr et al. (1993) and this was confirmed in the present study (20% at 80 µM) (Fuhr et al., 1993). However, extract B contained naringenin glucosides only at about 8 µM in total. Even if the glucosides were as potent as the aglycone of naringenin, this concentration would have been too low to inhibit CYP1A2. Taken together, at present, too little information is available to explain the observed differences between the extracts or to attribute the effects to individual extract constituents. Both salicylates and flavonoids as well as other phenolic compounds, such as syrengin, or yet unidentified compounds in the extracts and possible additivity between the compounds need to be investigated with respect to CYP450 inhibition and their role further elucidated in the future.

From the about 450 *Salix* species which are known (Lauron-Moreau et al., 2015), only few of them are of medical interest so far according to the guidelines of EMA and the United States Pharmacopeia (EMA, 2017; Oketch-Rabah et al., 2019). However, *Salix* species show huge differences in their phytochemical content, depending on the genotype (Förster et al., 2008; Förster et al., 2010; Gawlik-Dziki et al., 2014; Gligorić et al., 2019), and also other factors such as the plant part used as a source material for medical products (Gawlik-Dziki et al., 2014; Sugier et al., 2013). This currently complicates a reliable therapeutic efficacy of the product. Based on the present data, it also calls for further systematic and more detailed studies on possible drug interactions. For the moment, a potential interaction with drugs that are metabolized by CYP2C19, CYP1A2, as well as CYP3A4 with *Salix* cortex containing formulations cannot be excluded in common dosages. Although after oral intake, the amount of most phytochemicals in *Salix* species that becomes accessible for absorption through the epithelial layer of the gastrointestinal tract is currently not known (Schmid et al., 2001), it must be considered that e.g., CYP3A4 is not only the most abundant CYP in the liver but also the wall of the small intestine. There, before absorption into the blood stream occurs, it plays a major role in the metabolism of many different drugs such as calcium channel blocker, lovastatin or diazepam (Vuppalanchi and Saxena, 2011), which either limits or increases the amount of bioavailable active drug. Especially people that have an inherent risk of polypharmacy and consider long-term use of *Salix* products (ESCOP, 2017) should be aware of this.

## Data Availability

The original contributions presented in the study are included in the article, further inquiries can be directed to the corresponding author.
